# A Sensor Fusion Method Using Transfer Learning Models for Equipment Condition Monitoring

**DOI:** 10.3390/s22186791

**Published:** 2022-09-08

**Authors:** Eyup Cinar

**Affiliations:** 1 Department of Computer Engineering, Eskisehir Osmangazi University, Eskisehir 26040, Turkey; eyup.cinar@ogu.edu.tr; 2 Center of Intelligent Systems Applications and Research (CISAR), Eskisehir Osmangazi University, Eskisehir 26040, Turkey

**Keywords:** sensor fusion, deep learning, transfer learning, condition monitoring, bearing fault diagnosis

## Abstract

Sensor fusion is becoming increasingly popular in condition monitoring. Many studies rely on a fusion-level strategy to enable the most effective decision-making and improve classification accuracy. Most studies rely on feature-level fusion with a custom-built deep learning architecture. However, this may limit the ability to use the widely available pre-trained deep learning architectures available to users today. This study proposes a new method for sensor fusion based on concepts inspired by image fusion. The method enables the fusion of multiple and heterogeneous sensors in the time-frequency domain by fusing spectrogram images. The method’s effectiveness is tested with transfer learning (TL) techniques on four different pre-trained convolutional neural network (CNN) based model architectures using an original test environment and data acquisition system. The results show that the proposed sensor fusion technique effectively classifies device faults and the pre-trained TL models enrich the model training capabilities.

## 1. Introduction

Industry 4.0 has brought innovations and perspectives into conventional manufacturing processes. A transition from automation to autonomy with an aim to enhance efficiency and reduce cost has become possible by implementing smart systems and algorithms. While cyber-physical systems (CPS) play a vital role in today’s manufacturing facilities with the help of the Internet of Things (IoT) and sensor technologies, the connected manufacturing equipment in a CPS can generate a huge amount of data every day that is disposed of. However, the new trend is to make the most out of sensor data and integrate it with traditional manufacturing execution systems. In this case, computational models for IoT system-generated data can add a significant value [1]. This might find important applications, especially in fault detection and equipment maintenance. For this reason, data-driven applications for condition monitoring and intelligent fault detection are becoming increasingly popular. Using machine learning (ML) and deep learning (DL) algorithms, sensor data is fed as input to these algorithms to map fault fingerprints into actual fault classes. Because these ML and DL models can excel at detecting fault patterns better than human operators, they can generate early warnings of potential equipment failures.

Many studies in the literature address the development of more efficient models by designing new DL networks for equipment condition monitoring and fault detection applications, e.g., refs. [2,3,4]. In addition to selecting the most appropriate model, current studies also focus on strategies that can incorporate information sources from multiple sensors to improve fault diagnosis performance. These studies can also find important applications in structural health monitoring [5]. The multiple sources of different types of sensor data, whether coming from physical equipment or its digital replica by utilizing Digital Twin concepts [6], can be used to detect or model anomalies before they occur. Under the umbrella term “sensor fusion”, various strategies can be used to combine information from multiple sensors. These strategies can be grouped into three main categories in terms of the level of data processing abstraction. These are sensor fusion strategies at the data level, the feature level, and the decision level. [6]. As the name implies, data-level fusion combines sensor data at an early stage. Feature-level strategies rely on preprocessing of data and first use feature extraction methods. The extracted features are combined into a new set of features that can then be provided to various machine learning algorithms for classification tasks. Finally, decision-level strategies rely on aggregating the decisions made by individual sensors to reach a consensus. The communication overhead and processing complexity can be high for a data-level fusion but information loss is usually avoided. Conversely, decision-level fusion results in lower communication load and processing complexity however can yield to information loss [6].

Xia et al. [7] reported superior monitoring and fault diagnosis performance when their sensor fusion technique was applied to their case study. In their study, multi-channel sensor data with the same type of sensors were fused into a higher-order tensor array, and designed features were extracted from the array to monitor statistically for out-of-control cases to detect process faults. The technique is limited to the same type of sensor data fusion and also requires a designed feature extraction step. Suawa et al. [8] presented a sensor-fusion study and integrated different types of multi-sensor data, utilizing data-level fusion. Sound and vibration sensors were utilized to detect brushless DC motor faults utilizing deep learning (DL) models such as deep convolution neural networks (DCNN) and the long-short-term memory method (LSTM). The results show an improved accuracy in fault classification when sensor fusion is employed. However, since the proposed technique only considers data fusion in the time domain using raw data, it is not possible to integrate frequency domain information into the fusion. A similar drawback also exists in the study Huang and Lee proposed in ref. [9]. They utilized raw data obtained from multiple types of sensors, extracted features by utilizing convolutional operators frequently used in CNNs, and then performed feature-level fusion to estimate tool wear or surface roughness. A technique that relates both image and sensor fusion was proposed by Wang et al. [10], where raw vibrational data was converted into grayscale images, and sensor channel data were concatenated to form a grayscale composite image. The method is limited to only a single type of sensor and, more importantly, does not cover information from the frequency domain, which is critical for signals such as vibration. Furthermore, the concatenation of channel segments changes the length of image size every time a new sensor channel is added, which can hinder the utilization of a pretrained DL model. Wang et al. [11] proposed a DL-based data fusion approach where both time and frequency domain transformed data can be integrated into a feature-level fusion strategy. The authors also introduced attention-based enhancements into the DL algorithm and claimed that attention and feature level fusion increase the classification accuracy in a motor-bearing fault diagnosis classification study. The proposed technique allows the integration of multi-type sensor data and time and frequency information fusion from sensors. However, feature-level fusion locks in the user utilizing only this custom-mode algorithm design and does not allow utilization of any further models, which might be critical for verification on different deep learning model alternatives. 

Sensor fusion techniques also find important applications in image processing [12]. Images acquired from sensors can be merged to integrate more information into a single image. Fusing images can be achieved in different techniques, which can be classified as pixel level, decision level, and feature level fusion [13]. Pixel-level techniques that take place under spatial domain techniques are among the less computationally expensive ones. They simply rely on selecting among different corresponding pixel values of multiple image data, based on a mathematical formula or an algorithm. Successful implementations of pixel-level image fusion especially stand out in medical image processing [14]. In addition, the fusion of computed tomography images obtained by multi-modal sensors such as optical and acoustic emissions is demonstrated to significantly improve automated defect recognition for additive manufacturing [15]. One of the main motivations of this study is to demonstrate an application of image fusion in a fault diagnosis domain for the first time.

Although DL models and sensor fusion techniques are reported to present superior performance in terms of classification accuracy, there is still a requirement for a large amount of data for DL models. Especially when dealing with fault diagnosis problems, this might be a bottleneck since the actual fault data might not be available abundantly; therefore, training on a smaller dataset should be possible. In order to address this issue, researchers studied transfer learning techniques [16]. In the machine learning (ML) field, transfer learning (TL) is a method to transfer the generalized knowledge gained from prior classification experiences into different but related domains. Transfer learning relies on re-using a pre-trained model in a different experiment to solve a different problem. The power advantages include significantly reduced training data, computing resources, and training time [17]. Liu et al. [18] studied transfer learning capabilities using CNN models for a building chiller energy system fault diagnosis scenario. Based on their results by utilizing transfer learning with less data, maximum accuracy improvements up to 12.63% were obtained. Mao et al. [19] utilized a CNN-based model called VGG-16 that was pre-trained on a large-scale image dataset called ImageNET to demonstrate the effectiveness of TL in fault diagnosis of bearing faults. Since the pretrained network model enhances the VGG-16 model’s image feature learning ability on images, the trained model exhibits better performance on fault diagnosis tasks. Instead of utilizing raw data, Lee et al. [20] studied obtaining short-time-Fourier-transform (STFT) images of single-axis vibration channels and compared pretrained DL network models such as VGG-19, SquezeeNet, and AlexNet in the characterization of defects for complex machinery called gravity acceleration equipment. The results suggest superior classification performance as compared to traditional ML models. Similar to other studies, despite leveraging TL advantages in the study, the lack of sensor fusion capability can be counted as a major disadvantage. 

This study demonstrates a new sensor fusion technique that can be readily utilized in equipment fault diagnosis applications with heterogenous multi-modal sensors utilizing a fusion of spectrogram images into a single image. The technique can incorporate both time and frequency domain information simultaneously and can easily be adapted for transfer learning tasks with a resultant fixed-size fused image spectrogram. The fusion technique is an expansion of the author’s early preliminary work on a single type of sensor fusion [21] and is inspired by traditional image fusion methodologies in the literature; however, it has not been employed before in fault diagnosis applications with heterogeneous sensors. Furthermore, in this study, from the perspective of DL-based data-driven fault diagnosis solutions, TL techniques are employed and leveraged in terms of data and computational efficiency advantages. In order to test the effectiveness of the proposed method, an induction motor’s fault use case is utilized since they are the most commonly used equipment in manufacturing. Monitoring and detecting the faults of induction motors at early stages are important for continuous production lines and labor-saving. The faults of induction motors may be classified as electrical and mechanical. The electrical faults include rotor and stator faults, while the mechanical faults consist of eccentricity faults, bearing faults, and misalignment faults.

The remainder of the article is organized as follows: Section 2 introduces the reader to related theories and background information. Then, in Section 3, the details of the proposed sensor fusion are presented. Section 4 presents the experimental setup and data acquisition system used in the experimental verification of the proposed method. Section 5 presents the results of the experimental tests, followed by Section 6. Finally, the Section 7 is presented.

## 2. Theory and Background

In this section, the reader is introduced to related theories and background information. First, the short-time Fourier transform is introduced, and then the basics of the specific deep learning architectures used in the experiments are detailed.

### 2.1. Short-Time Fourier Transform

Different preprocessing methods can be used when processing signals received from sensors for ML depending on the type of signals. These can be used either in the time domain or in the frequency domain. The original time series data are transformed into a frequency domain representation by applying transformation methods such as the Fourier transform. However, the time component is no longer available in the Fourier transform signal representation. The short-time Fourier transform (*STFT*) can be used to include time-varying frequency changes in the results. In particular, for signals originating from non-stationary processes, the *STFT* could contain more information. The transformed data can be converted into a two-dimensional time-frequency image called a spectrogram. The transformation formula is provided in Equation (1).

(1) $$STFT_{x}\left(\tau ,f\right)=\int_{-\infty }^{+\infty }x\left(t\right)h\left(t-\tau \right)\exp(-j2\pi ft)dt,$$

where *x*(*t*) is the original time-domain signal and ℎ(*t* − *τ*) is a windowing function centered at time *τ*. Therefore, the segmented signal is *x*(*t*)ℎ(*t* − *τ*). In short, ℎ(*t* − *τ*)exp(-*j*2π*ft*) is the basis of the *STFT* function that transforms the time domain input signals into time-frequency representations.

### 2.2. Convolutional Neural Networks

Convolutional neural networks (CNNs) are neural networks with a special operator called convolution. In general, the raw input data at the beginning of the neural network is processed and sampled with multi-layer cascaded convolutional filters and pooling operations to extract discriminative features that facilitate the detection of patterns in the input data. Since its first name originated from the design of Lecun et al. [22] and is now famously known as LeNet-5, many researchers have proposed different types of CNNs tailored to specific needs in the application domain. Although the various CNN architectures may have slight differences specific to the type of network, the basic concept of convolution and pooling mechanisms is common to all. The common elements for a CNN include: convolutional layers, pooling layers, and fully connected layers.

Convolutional Layers 

These types of layers are generally placed at the beginning of a multi-layer CNN and include convolution operators onto input data. A convolution layer can include a filter (kernel) of one or multi-dimensional size, and kernel slides over the input matrix data to generate a feature map. The amount of slides is an important parameter called stride. If an input is a tensor, it can have a conv kernel shape of *n x h x w x c*, where *n* is the number of inputs, *h* is the height of the feature map, *w* is the width of the feature map, and *c* is the number of channels for the feature map. The size of a convolution kernel is a critical parameter, and if the kernel size is incompatible with the input data, then the data can be pad with artificial input such as zeros. This is called a padding operation. The resulting data, called the feature map, is usually passed through an activation layer to introduce nonlinearity with specific activation functions. The most common activation functions in CNNs are ReLu, Tanh, and Sigmoid functions. After the activation layer resulting feature map is usually passed over a pooling layer.

Pooling Layers 

Pooling layers are utilized to reduce the size of feature map data but retain the important information in a feature map to be passed over to the next layer. As the pooling kernel with a kernel size of *n x m* slides over a feature map data, an arithmetic operation such as maximum or average is applied. The resultant feature map is then transferred into the next layer in the network.

Fully Connected Layers 

This layer is a standard traditional neural network layer that can be found in multi-layer artificial neural networks. It is most commonly utilized through the end of the CNN models to map resultant feature maps into target classes.

Background on the Types of CNN Models Utilized 

This section provides background information on the specific types of CNN models employed in this study for comparison.

VGG-16 Model 

Proposed by Simonyan and Zisserman [23] from the Visual Geometry Group (VGG) at the University of Oxford, VGG-16 has a total of 16 learnable weight layers and relies on 13 stacks of convolutional networks (ConvNet) with 3 × 3 filter sizes. The last three layers include fully connected layers. The network is designed to accept an RGB color image input size of 224 × 224 and has 138 million parameters in terms of the total network number of parameters.

AlexNet 

This type of CNN proposed by Alex et al. [24] has eight layers with a total of 61 million parameters. The initial five layers include convolutional layers and end with three fully connected layers. AlexNet is configured to accept an RGB color input image size of 227 × 227.

GoogLeNet Model 

GoogLeNet is a type of CNN with 22 layers of depth, and it inherits fundamental parts of an earlier version of a network called the inception network [25]. GoogLeNet accepts RGB color images with an input size of 224 × 224 and has 7 million parameters in total.

SqeezeNet Model 

**Figure 1:**
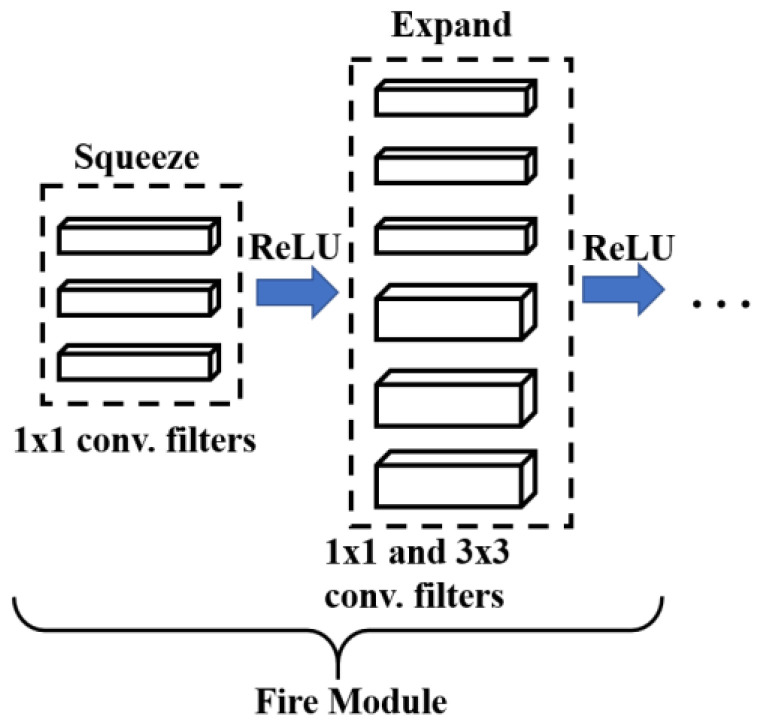
The fire module in Squzeenet micro-architectural overview.

Iandola et al. [26] proposed a network called SquezeeNet with the least number of parameters and smaller model size for more efficient run-time inference performance, claiming not to jeopardize the classification performance. Figure 1 show the fundamental micro architecture module belonging to SquezeeNet called the fire module. The macro-architecture involves cascaded fire modules and a few more convolution and pooling layers 18 layers deep. The size of the feature maps is reduced by reducing the size of the convolutional kernels (to 1D filters) via the fire module. The squeeze module reduces the input dimension by utilizing 1 × 1 filters, and in the expanding layer, a portion of the filter is replaced with 1 × 1 convolutions instead of only 3 × 3 filters. Note that since the major computational operations in CNNs are convolutional operations, the smaller the filter size, the better the computational efficiency.

### 2.3. Transfer Learning

Transfer learning is a machine learning (ML) method to improve model performance by transferring knowledge from the same or related source domains into a target domain [27]. Instead of training a model from scratch, a pretrained model can be incorporated to improve a particular target task.

For a formal definition: Let D define a domain for an ML classification problem that consists of two parts D={X, P(X)}, where X represents the feature space and P(X) represents a marginal probability distribution. A feature vector $x=\left\{x_{1,\;}\;\ldots ,\;x_{n\;}\right\}\;\in X$ is a particular element of the feature space, and y is the corresponding class label belonging to the label space Y. For a domain D, a task can be defined as T={Y,  f(.)}, where f(.) is the predictive function learned from pairs $\left\{x_{i,\;}\;y_{i}\right\}$. A source domain dataset can be defined as $D_{s}=\left\{\;\left(x_{s1},\;\;y_{s1}\right),\;\ldots \left(x_{sn},\;\;y_{sn}\right)\right\},$ where $\;x_{s1}\;\in \;X_{s}$ of $D_{s}$ and $y_{si}\;\in \;Y_{s}$ for the corresponding class label. Similarly, a target domain can be defined as $D_{T}=\left\{\;\left(x_{T1},\;\;y_{T1}\right),\;\ldots \left(x_{Tn},\;\;y_{Tn}\right)\right\}$. The tasks for source and target domains are $T_{s}$ and $T_{T},$ respectively. If the predictive functions for the source and target tasks are $f_{s}\left(.\right)$ and $f_{T}\left(.\right)$ respectively then transfer learning can be formally defined to improve $f_{T}\left(.\right)$ by utilizing knowledge obtained from $D_{s}$ and $T_{s}$ when $D_{s}\;\neq \;D_{T}$ or $T_{s}\;\neq \;T_{T}$.

In this paper $D_{s}$ and $T_{s}$ are obtained utilizing the popular ImageNet dataset and trained for classification of images utilizing various CNN model types. The source model optimized to recognize features from over a million images aims to contribute to the target domain spectrogram image classification task. 

## 3. The Proposed Sensor Fusion Method

The proposed method involves two steps. The first is the conversion of the sensor signals into a time-frequency domain using the STFT transform and the generation of RGB images from the raw sensor data. The second involves the use of transfer learning using multiple CNN-based deep learning models.

**Figure 2:**
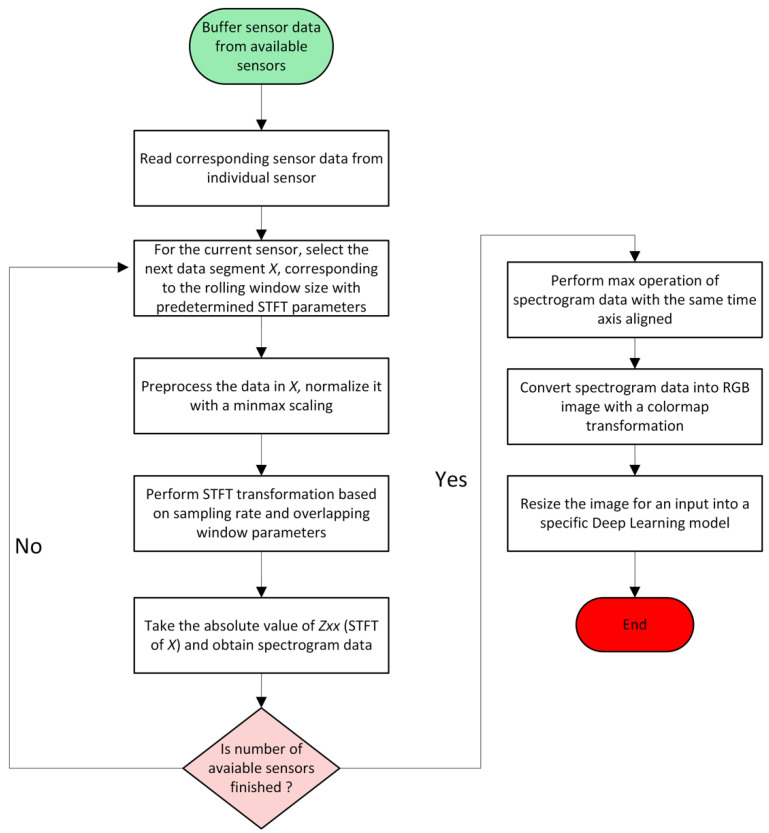
STFT image transformation flow chart detailing a step-by-step of the proposed sensor fusion algorithm.

Figure 2 shows a flowchart detailing the conversion of raw sensor signal data into spectrogram images and the final fusion technique for multi-sensor fusion. First, the sensor data coming from multiple sensors are buffered and read as rolling window segments. For each data segment, the data are normalized with min-max scaling and the STFT transform is extracted based on the sampling frequency. A spectrogram is obtained by taking the time values of the segments and the absolute values of the Fourier transformed values. When all the buffered sensor data are finished, a maximum operation is performed over the spectrogram data, and multiple spectrogram data are mapped into a fused spectrogram with a time segment alignment of the sensors. The final global spectrogram of the data segment is converted into an RGB image by mapping the spectrogram grid values with a color map. Finally, the resulting image is resized based on the expected image input size of the deep learning target architecture.

**Figure 3:**
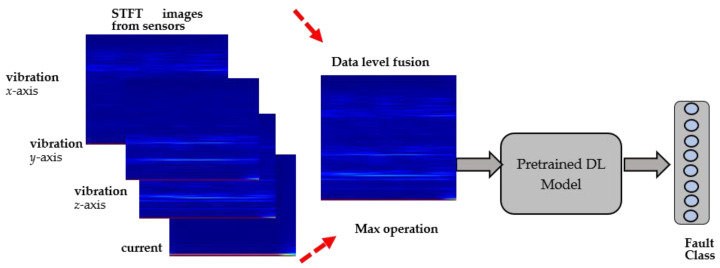
An illustrative figure representing the proposed sensor fusion method.

Figure 3 illustrate the proposed sensor fusion technique in detail. Each spectrogram image corresponds to individual sensors, such as vibrational axes x, y, and z, and the current sensor. Each spectrogram image is illustrated at the beginning. Then, spectral images are fused into one global image and resized according to the specific transfer learning model’s image input size.

The explored pretrained models include VGG-16, AlexNet, GoogleNet, and SqueezeNet. All models are pretrained on ImageNet [22] dataset’s images and frozen before starting the training process with the fused sensor images. Only the final fully connected layer with classification output is modified to match the number of fault classes in our experiments.

## 4. Experiments

This section describes the experimental setup used to validate the proposed method. A custom-built testbed is set up to generate artificial faults. The testbed setup is used to collect multisensory data through an IoT data pipeline into databases on a GPU server. Data processing, model training, and testing are performed on the test case scenario data.

### 4.1. Experimental Setup and Data Collection 

**Figure 4:**
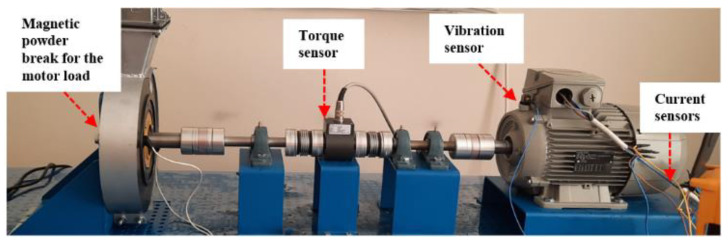
The testbed which is designed for generating artificially induced motor equipment faults.

**Figure 5:**
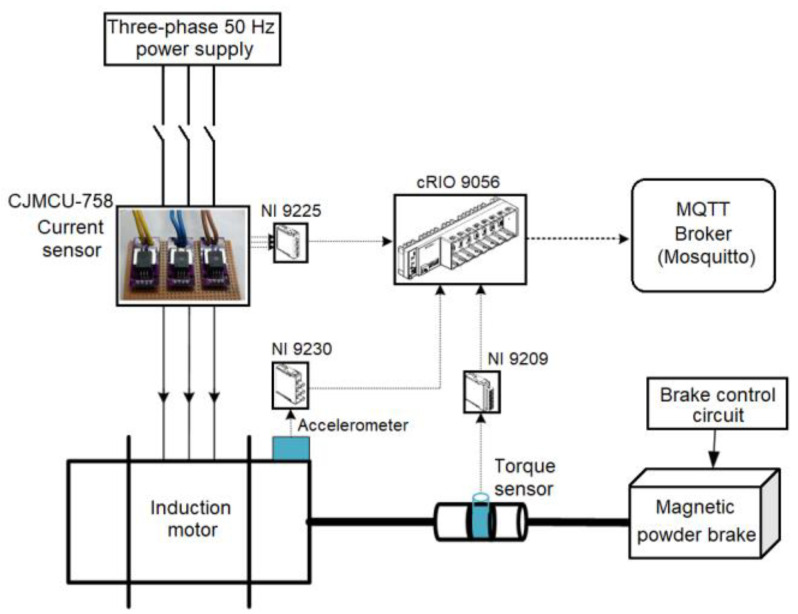
A schematic diagram of the experimental testbed designed and utilized in this study.

Fault conditions in an electrical motor’s operations are experimentally created in a controlled testbed setup designed for this research, as shown in Figure 4. Current, vibration, and torque signals are collected with a National Instrument (NI) data acquisition system (CompactRio 9530). A three-axis accelerometer sensor (PCB triaxial Model 356A15) placed on the front end of the motor independently measures the three-channel (x, y, z) vibration during the motor’s operation under varying load conditions. Figure 5 show a schematic diagram illustrating high-level connections of the main components on the manufactured testbed.

### 4.2. Fault Induced Scenarios 

**Figure 6:**
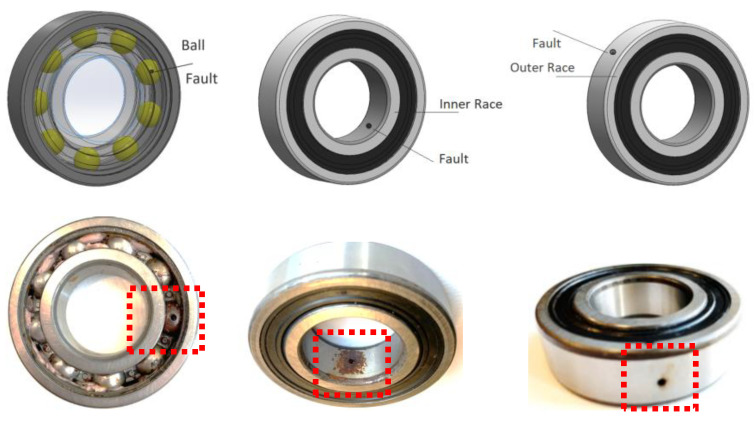
The real-time multisensory fault diagnosis.

A total of eight different test conditions are artificially created on the motor’s bearing parts. Figure 6 demonstrate some faults created on the motor’s actual bearing part. The four leading groups of the classes include healthy, inner race fault, outer race fault, and ball bearing fault types. The fault types also vary in terms of the depth of the holes opened on bearings intended to indicate a level of fault severity. The depth of the holes range from 0.5 mm to 2.5 mm. The opening of artificial holes is created by utilizing a high-precision arc cutting machine applied through the corresponding part of the fault surface of the bearings.

**Figure 7:**
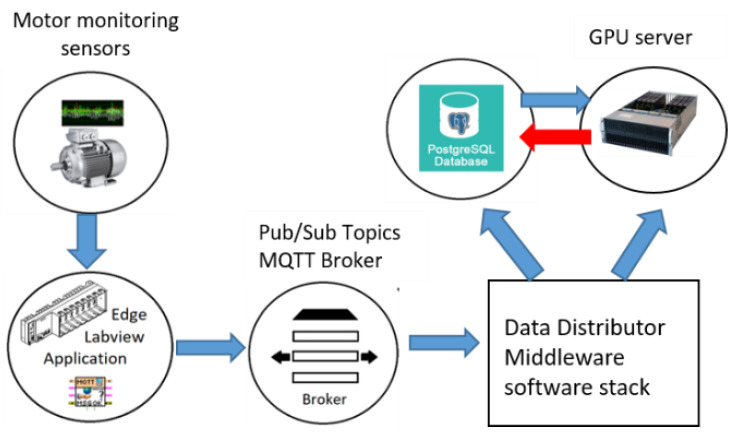
An IoT data pipeline utilized to collect data from the testbed and store sensor data in databases on a GPU server.

For online data collection, an end-to-end data pipeline is designed and implemented. Figure 7 illustrate the main components of the architecture. The data acquisition module collects and buffers sensor data in memory on temporary local storage files. MQTT data transfer protocol transports data in topics via pub/sub protocol. A separate topic is created for each sensor type, and an MQTT client that runs in edge devices such as cRIO publishes the sensor data in batches through the topics on the MQTT broker program called Mosquitto broker. After the current batch is posted to the broker, the data are wiped programmatically to free up the space for upcoming motor operations data collection.

The broker service sends data to a central middleware data distributor service. The middleware acts as a data distributor and forwarder between various other services. It ensures that the data is transferred to long-term data persistence services without data loss. Furthermore, it allows the data pipeline to be scalable to a fleet of equipment for data collection and monitoring in the future. A database system called PostgreSQL stores the data with a global timestamp. Timestamp info is created at the DAQ edge layer and appended to the data packets with the metadata for session information, such as the fault type label of the experiment. A GPU server connected to the middleware software system and the database can query the data, monitor the equipment sensor status, and process the historical data for the model training. It can also post to the database tables for results to be updated and tracked through a data analytics dashboard.

Experimental data storage, signal processing, model training, and testing are performed on a server computer (Supermicro, 64 GB computer RAM with two NVIDIA RTX 3080 GPUs) utilizing Python for data retrieval, preprocessing, and MATLAB for the transfer learning model training and testing operations.

Each experimental data collection session takes 40 seconds and is stored in the database with the corresponding fault label. The data stream collected from each sensor’s experiments is sampled at a 6400 Hz sampling rate with a CompactRIO edge device-appropriate hardware modules.

## 5. Results

**Figure 8:**
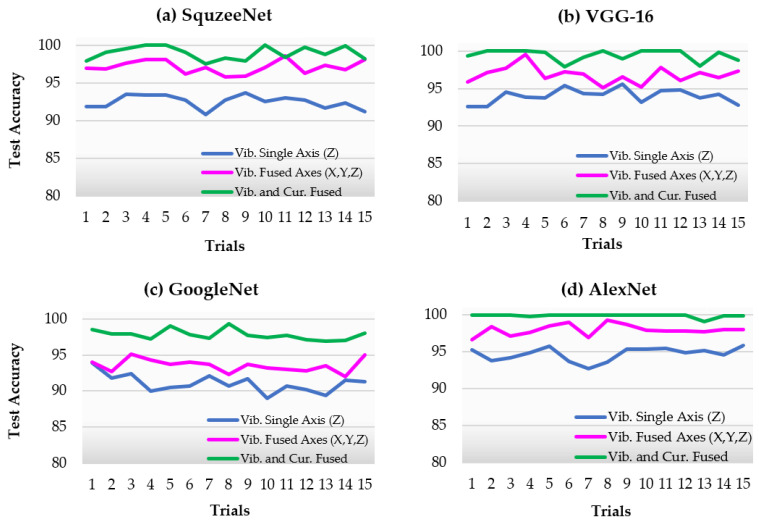
Testing accuracy versus experimental trials performed for each deep neural network model.

Figure 8 show the percent test accuracy obtained with the trained model in 15 trials using four different deep learning model architectures. The green line represents the classification accuracy when all sensor information is fused. This includes the three axes of the vibration channel (*X, Y*, and *Z*) and the motor current sensor. The purple line indicates that only the vibration sensor channels are fused using the proposed method. The blue lines show the single vibration axis *Z* used for the model.

A Python script was written to read the corresponding sensor data window range from the database and bring it into the script’s memory for further processing to generate STFT images and save them to the server’s hard drive in JPEG format. Each experimental data set included a total of 1500 spectrogram image samples. Of these samples, 20% of the data was left for testing and isolated. The remaining dataset was used to train the model (60%), and the rest was left to validate the model during training. The selection of training and validation data was carried out randomly for each experimental trial and model training session. Model training and testing were performed using MATLAB 2021 and two NVIDIA RTX 3080 GPU cards on the GPU server. After the training sessions were completed, the models were run for inference with the isolated testing data, and percent testing accuracies were calculated and charted, as shown in Figure 8. 

All weights of the deep learning model were transferred from the models which were previously optimized and pretrained utilizing the famous ImageNet dataset. The model training incorporated the fused spectrogram images on top of the previously trained network. All of the models were trained with the same hyperparameters. These include stochastic gradient descent with momentum as the optimizer, a momentum of 0.9, a mini-batch size of 10, an initial learning rate of 3 × 10^−4^ with a learning rate drop factor of 0.1, and a validation frequency of 15 epochs. The maximum number of epochs was limited to 50. Early termination was executed if no further progress was obtained during the training to avoid overtraining. 

**Figure 9:**
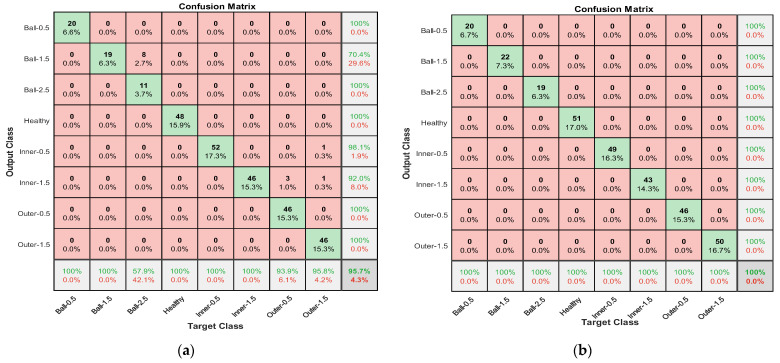
The two confusion matrices belonging to (**a**) GoogleNet and (**b**) AlexNet when the proposed data fusion is applied to all of the available sensors.

In terms of average testing classification accuracies when all sensor information was fused (green line), GoogleNet showed a relatively lower average accuracy with 97.807 %, whereas AlexNet showed the highest, 99.90%, with less deviation. Figure 9 present the confusion matrices belonging to these two separate models.

## 6. Discussions

In this paper, the proposed sensor fusion method was tested with four deep learning architectures using transfer learning capabilities. The results show that the proposed sensor fusion method is effective and can help improve classification performance compared to using single sensor data for equipment fault detection and classification.

Analyzing the accuracy results of four different architectures in Figure 8, it can be seen that AlexNet (8 layers) has relatively better and more robust classification accuracy than another deeper network architecture, GoogleNet (22 layers). These results are also comparable to other transfer learning studies published in the literature. Bressem et al. [17] claimed that for the 16 different CNN architectures reinforced with transfer learning for chest x-ray image classification, the shallow networks perform comparably or better than their more complex counterparts with shorter training times. The reason could be the vanishing gradient problem. As a deep learning network becomes deeper, the initial layers tend to become stuck when updating the weights, resulting in no learning.

Achieving comparable classification performance with shallow networks is even more encouraging when considering the memory requirements of the models. Comparing VGG-16 (13 conv + 3 fully connected layers), which consists of 138 million parameters, to AlexNet with 61 million parameters, employing shallower networks results in significantly lower memory requirements (AlexNet model size: 227 MB, whereas VGG-16 is 515 MB).

Several experiments were also performed on the models where all pre-trained weights obtained from the ImageNet dataset were reset, and the network was configured to learn the weights from scratch using the same dataset size. In most cases, the network did not appear to converge or overtraining occurred. This shows the great benefit of transfer learning. Since the pre-trained network already has the ability to recognize features from the images in the ImageNet dataset, this ability seemed to help positively recognize fused spectrogram images.

## 7. Conclusions

This paper proposed a new sensor fusion method in which multisensory information can be fused in the time-frequency domain. The effectiveness of the proposed method was verified using a use case for equipment fault detection and classification. In addition, a new testbed design and data acquisition system was demonstrated. Artificially induced motor bearing fault scenarios were created, and data were collected through an IoT data pipeline.

Transfer learning was leveraged in the proposed sensor fusion technique, and four different CNN-based deep learning architectures were employed. The results show that the proposed sensor fusion technique is effective and can be used to detect and classify equipment faults for predictive maintenance applications.

Future research includes expanding the proposed method into additional motor faults such as stator, misalignment, or winding faults. Furthermore, incorporating additional sensors such as torque or electromagnetic sensors will also be considered. Lastly, transferring the trained models for an embedded edge-AI real-time application in the field is among the future goals of this research. 

## Data Availability

Not applicable.
